# MADS-box Transcription Factor *OsMADS25* Regulates Root Development through Affection of Nitrate Accumulation in Rice

**DOI:** 10.1371/journal.pone.0135196

**Published:** 2015-08-10

**Authors:** Chunyan Yu, Yihua Liu, Aidong Zhang, Sha Su, An Yan, Linli Huang, Imran Ali, Yu Liu, Brian G. Forde, Yinbo Gan

**Affiliations:** 1 Zhejiang Key Lab of Crop Germplasm, Department of Agronomy, College of Agriculture and Biotechnology, Zhejiang University, Hangzhou, China; 2 College of Life Sciences, Zhejiang University, Hangzhou, China; 3 Centre for Sustainable Agriculture, Lancaster Environment Centre, Lancaster University, Lancaster, United Kingdom; Institute of Genetics and Developmental Biology, Chinese Academy of Sciences, CHINA

## Abstract

MADS-box transcription factors are vital regulators participating in plant growth and development process and the functions of most of them are still unknown. *ANR1* was reported to play a key role in controlling lateral root development through nitrate signal in *Arabidopsis*. *OsMADS25* is one of five *ANR1*-like genes in *Oryza Sativa* and belongs to the *ANR1* clade. Here we have investigated the role of *OsMADS25* in the plant’s responses to external nitrate in *Oryza Sativa*. Our results showed that OsMADS25 protein was found in the nucleus as well as in the cytoplasm. Over-expression of *OsMADS25* significantly promoted lateral and primary root growth as well as shoot growth in a nitrate-dependent manner in *Arabidopsis*. *OsMADS25* overexpression in transgenic rice resulted in significantly increased primary root length, lateral root number, lateral root length and shoot fresh weight in the presence of nitrate. Down-regulation of *OsMADS25* in transgenic rice exhibited significantly reduced shoot and root growth in the presence of nitrate. Furthermore, over-expression of *OsMADS25* in transgenic rice promoted nitrate accumulation and significantly increased the expressions of nitrate transporter genes at high rates of nitrate supply while down-regulation of *OsMADS25* produced the opposite effect. Taken together, our findings suggest that *OsMADS25* is a positive regulator control lateral and primary root development in rice.

## Introduction

Nitrogen (N) is one of the essential macronutrients required by plants for normal growth and development and is frequently the major limiting factor for crop yields [1]. For higher plants the major source of N is usually in the form of nitrate (NO_3_^-^) [2, 3]. One of the most important functions of NO_3_^-^ is to provide nitrogen for synthesis of amino acids and other forms of organic N [4]. In addition, NO_3_^-^ acts as a signal to regulate many metabolic and developmental processes such as transcription and translation, energy transfer, protein accumulation, cytokinin transport, seed germination, and plant growth and development [1, 5–8]. The NO_3_^-^ content of aerobic soils can vary markedly in time and space [2, 3, 9], requiring plants to evolve sophisticated signaling and transport processes to enable them to adjust to these variations.

Rice is one of the most important food crops and N deficiency is considered as an important limiting factor affecting its productivity [2, 3]. Although under flooded conditions rice mainly takes up N in the form of ammonium, NO_3_^-^ still contributes 15–40% of the total N absorbed by the rice crop [10, 11]. Furthermore, NO_3_^-^ enhances the uptake and assimilation of ammonium by rice plants [11]. In plants, NO_3_^-^ is mainly taken up by two mechanisms, namely the high affinity uptake system (HATS) and low affinity uptake system (LATS) [12]. It has been suggested that two families of membrane proteins, the nitrate transporter 1 /peptide transporter family (NRT1/PTR) and nitrate transporter 2 (NRT2), are involved in NO_3_^-^ uptake by plants [12–14]. NRT1/PTR family is named unified NPF according to the phylogenetic relationship of these proteins [15]. NRT2 proteins are the high affinity nitrate transporters while most of the NPF family is low affinity nitrate [16–19].

A properly developed root system is essential to ensure the optimum uptake of water and mineral nutrients by plants [20, 21]. As the key component of root system, lateral root (LR) initiation and development is affected by the combined actions of gene regulation, hormone and environmental signals such as light, water and nutrient [22–25]. In *Arabidopsis*, the *ANR1* and *AtABF3* genes are reported to be involved in distinct NO_3_^-^ signaling pathways regulating LR development [26, 27]. *AtNPF6*.*3* functions upstream of *ANR1* in regulating LR elongation, apparently in its role as a NO_3_^-^ sensor [28, 29]. Exogenous application of NO_3_^-^ has been reported to affect the expression of *ANR1*, a MADS-box transcription factor in regulating LR numbers and LR elongation [26]. *ANR1* expression is induced by nitrate deprivation and constitutive over-regulation of *ANR1* in roots of transgenic *Arabidopsis* increases LR growth while having no direct effect on LR density or primary root growth [30, 31]. The root phenotype of the *ANR1* overexpressing lines mainly depends on the presence of NO_3_^-^, suggesting that there other components involve in NO_3_^-^-dependent signaling pathway.

Although NO_3_^-^ regulation of LR development has been extensively studied in *Arabidopsis*, little is known about this process in rice. miR444 has been reported to target four *ANR1*-like homologous genes (*OsMADS23*, *OsMADS27a*, *OsMADS27b* and *OsMADS57*) to regulate root development in rice [32–36]. miR444a regulates the NO_3_^-^-signaling pathway in rice roots as well as regulating NO_3_^-^ accumulation and the response to phosphate starvation [37]. *OsMADS25* is one of the five *ANR1*-like homologues in rice [32, 33]. Previous studies reported that the expression of *OsMADS25* is significantly induced by NO_3_^-^, salt and osmotic stress [38, 39]. It was also reported that *OsMADS25* is active in the central cylinder of the root and respond to auxin treatment [39]. To gain further insight into the possible regulatory functions of *OsMADS25* in control root development in rice, we have investigated its regulatory role in root development through NO_3_^-^ regulation.

## Experimental Procedures

### Plant material and growth conditions

*Oryza sativa* L. cv. *Nipponbare* was used as the wild type for both physiological and genetic transformation experiments. Rice sterilization, growth conditions and measurements were performed according to our previously reported study [38].

For NO_3_^-^ treatments, rice plants were grown in hydroponic culture or on Gelzan plates with modified 1/2 Murashige and Skoog salts in which KNO_3_ and NH_4_NO_3_ were replaced by KCl or KNO_3_ [37, 40]. To prepare cultures of different nitrate concentration, 0 mM KNO_3_, 0.2 mM KNO_3_ and 10 mM KNO_3_ were added to N-free medium [37].

For NH_4_^+^ treatments, rice seedlings were germinated and grown on Gelzan plates with modified 1/2 Murashige and Skoog salts in which KNO_3_ and NH_4_NO_3_ were replaced by KCl or NH_4_Cl, respectively. To prepare cultures of different ammonium concentration, 0 mM NH_4_Cl, 0.5 mM NH_4_Cl and 5 mM NH_4_Cl were added to N-free medium [41].

To examine the NO_3_^-^ response of overexpressing *OsMADS25* lines in *Arabidopsis*, surface-sterilized seeds were sown in 10×10 cm rectangular Petri dishes on medium containing 1% agar, 0.6%(w/v) sucrose, 1/50×B5 salts and 1 mM KCl and 1 mM glutamine was used to replace the nitrogen source of KNO_3_ and (NH_4_)_2_SO_4_ [31]. 7-day-old seedlings were transferred to fresh plates containing (in addition to 1 mM glutamine): 0 mM KNO_3_, 0.2 mM KNO_3_, 2 mM KNO_3_ or 10 mM KNO_3_ as N source [31]. Images taken at growth intervals of 16 d (no NO_3_^-^), 14 d (0.2 and 2 mM NO_3_^-^), and 13 d (10 mM NO_3_^-^) were used to analyze different root parameters [26, 31]. To investigate whether overexpression of *OsMADS25* affected early seedling development, surface-sterilized seeds were sown in 10×10 cm rectangular Petri dishes including 0, 0.2, 2 or 10 mM KNO_3_ [31]. After 2 d at 4°C, the plates were kept vertically at 22°C under the 16 h-light/8 h-dark light regime. Primary root length and the length of the first lateral root emerged were determined from images taken at 6 d, 8 d, 10 d, 12 d and 14d after sowing.

### Gene constructs and generation of transgenic plants

For the overexpression construct, a full-length *OsMADS25* cDNA was PCR-amplified and digested with restriction enzymes *Sal* I and *Sma* I for cloning into the pSB130-actin-NOS vector (a generous gift of Jumin Tu, Zhejiang University, China). To construct the RNA interference (RNAi) vector, a 259-bp cDNA fragment of *OsMADS25* was amplified and inserted into the *Bam*H I and *Kpn* I sites (for the reverse insert) and the *Sac* I and *Spe* I sites (for the forward insert) in the pTCK303 vector [42]. These constructs were transformed into rice using *Agrobacterium tumefaciens* EHA105 as previously described [43]. All transgenic lines were first selected based on the expression level of *OsMADS25* and further confirmed by their phenotype.

To construct 35S::*OsMADS25* for transformation into *Arabidopsis*, the 684 bp of the ORF were amplified by PCR and digested with *Sal* I and *Not* I and cloned into pENTR-1A vector. Subsequently the construct was recombined into pH2GW7 using the Gateway ‘LR reaction’ [44]. The binary vector construct was introduced into *Agrobacterium* strain GV3101 and *Arabidopsis* Col-0 plants were transformed by employing the floral dip method [45].

For the OsMADS25::YFP fusion, the 684 bp of the ORF was amplified by PCR and introduced in frame, after the YFP reporter gene of the 35S-pCAMBIA1300-YFP vector (a generous gift of Jumin Tu, Zhejiang University, China). Then the prepared construct was introduced into *Agrobacterium tumefaciens* strain EHA105. The recombinant constructs and free YFP (under the control of the 35S promoter) were introduced into epidermal cells of tobacco (*Nicotiana tabacum*) leaves which expressed the red fluorescent protein (RFP)-H2B by agroinfiltration [46, 47], and the epithelial tissue was examined using a scanning confocal laser microscope (Zeiss LSM 510) with a filter set for YFP fluorescence (514 nm for excitation and 525 nm for emission) and for RFP-fusion proteins using 543 nm laser lines. Primers used in the assembly of these constructs are listed in Table 1.

**Table 1:** Primer sequences used in this study.

Primer name	Sense primer (5'->3')	Anti-sense primer (5'->3')
*OsMADS25*	CCAGCTCAAGCATGAAATCAA	AAAGTTGCCTGTTGTTGTGGTGT
*OsActin*	CTTCATAGGAATGGAAGCTGCGGGT	CGACCACCTTGATCTTCATGCTGCT
*OsMADS25-OE*	GCGGTCGACATGGGGAGAGGGAAGATTG	GCGCCATGGTTATTCATCTTCAACTT
*OsMADS25-Ri*	GCGACTAGTGGTACCAGAGGAAATCTCCAACTTCAC	GCGGAGCTCGGATCCGCTTCTGGTAACTTGCTCACTT
*OsMADS25-yfp*	GCGTCGACATGGGGAGAGGGAAGATTG	CGAGCTCTTATTCATCTTCAACTTCTTTTTGAC
*OsMADS25-AtOE*	GCGTCGACATGGGGAGAGGGAAGATTG	TTGCGGCCGCTTATTCATCTTCAACTTCTTTTTGAC

### qRT-PCR analysis

Total RNA was isolated using the RNAiso Plus reagent from rice roots and shoots according to the manufacturer’s instructions. The first-strand cDNA was synthesized using the Reverse Transcriptase M-MLV from 2 μg total RNA in a 25 μl reaction, and diluted 4-fold with RNase-free water. Quantitative real-time RT-PCR was performed using SYBR Premix Ex Taq II as described in previous study [38, 48]. Expression of *OsActin* (Os03g0718100) was used as a reference to normalize expression of the other genes. Three biological replicates were performed for each RT-PCR experiment. Primers used for qRT-PCR are listed in Table 1.

### Measurement of tissue nitrate concentrations

Nitrate concentration was determined according to the method previously reported [37, 49]. Samples of root and shoot tissue (~2 g) were collected and immersed in 10 ml deionized water and heated at 100°C for 20 min. After cooling to room temperature, deionized water was added to the suspension to a 25 ml final volume. The suspension was centrifuged at 7000 g for 15 min and 0.1 ml supernatant was mixed with 0.4 ml 5% (w/v) salicylic acid in concentrated H_2_SO_4_. After 20 min at room temperature, 9.5 ml 8% (w/v) NaOH was added slowly into the mixture and after cooling again to room temperature and the absorbance of the samples was measured for absorbance readings at 410 nm wave length.

### Statistics

The results were analyzed by means of ANOVA for significance by IBM SPSS Statistics 21. Student’s t-test was analyzed to evaluate the significant difference between treatments at the probability at either 5% (P<0.05 with significant level *) or at 1% (P<0.01 with significant level **) as we previously described [50, 51].

## Results

### Subcellular localization of OsMADS25

**Fig 1:**
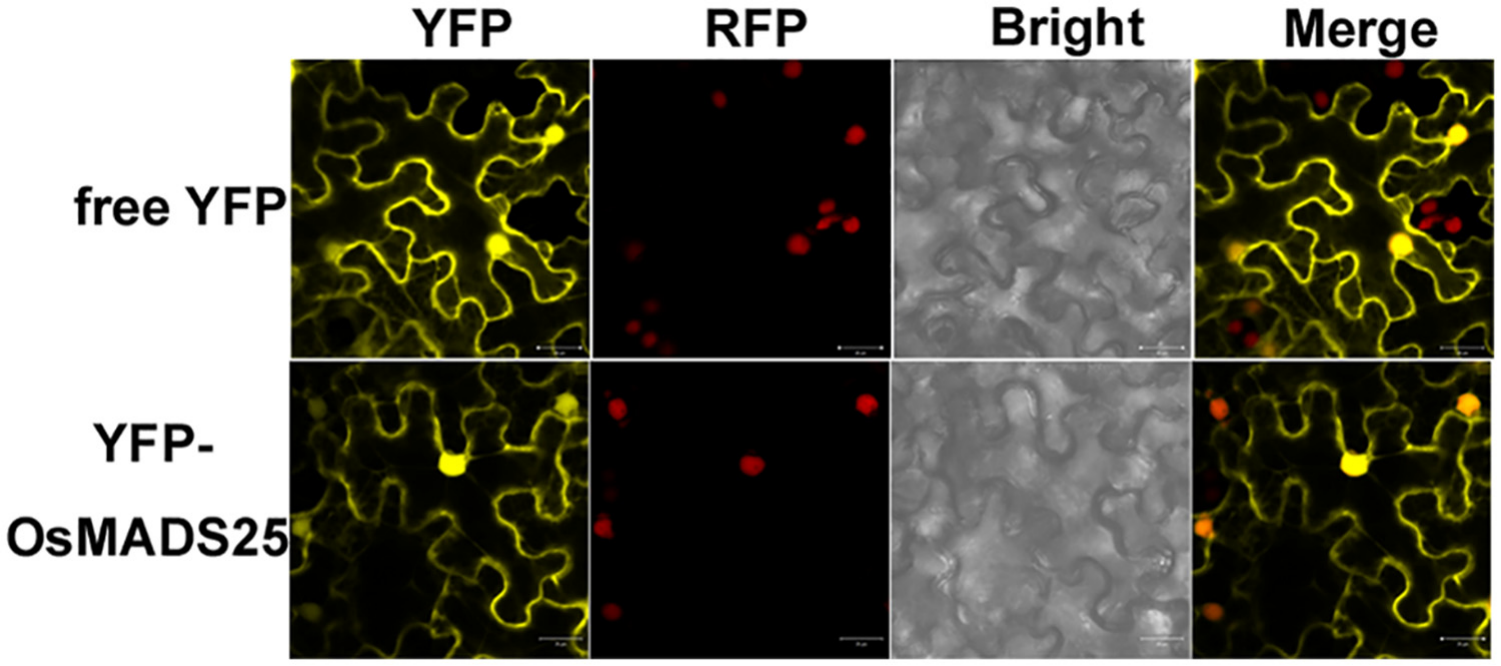
Subcellular localization of OsMADS25 protein. In the epidermal cells of *Nicotiana benthamiana* leaves expressing the RFP-fusion proteins, the YFP-OsMADS25 fusion protein and free YFP protein were transiently expressed. The bottom panels show the localization of YFP-OsMADS25 in tobacco epidermal cells in a transient assay, while upper panels show the localization of YFP as a control. Scale bars (20 μm) are shown. At least two individual experiments were performed for each combination with the similar results.

To investigate the intracellular localization of OsMADS25, its cDNA sequence was fused to the coding sequence of yellow fluorescent protein (YFP) under the control of the 35S promoter. The 35S:: YFP:: OsMADS25 construct and free YFP (under the control of the 35S promoter) alone were expressed in tobacco epidermal leaf cells which expressing RFP:H2B nuclear marker. The yellow fluorescent signals from the free YFP and YFP:: OsMADS25 were overlapped with the red fluorescent signal from the RFP-H2B (Fig 1). The confocal images in Fig 1, showed that fluorescence associated with expression of the YFP-OsMADS25 fusion protein was detected in both nucleus and cytoplasm.

### Effect of *OsMADS25*-overexpression on root development in *Arabidopsis*

**Fig 2:**
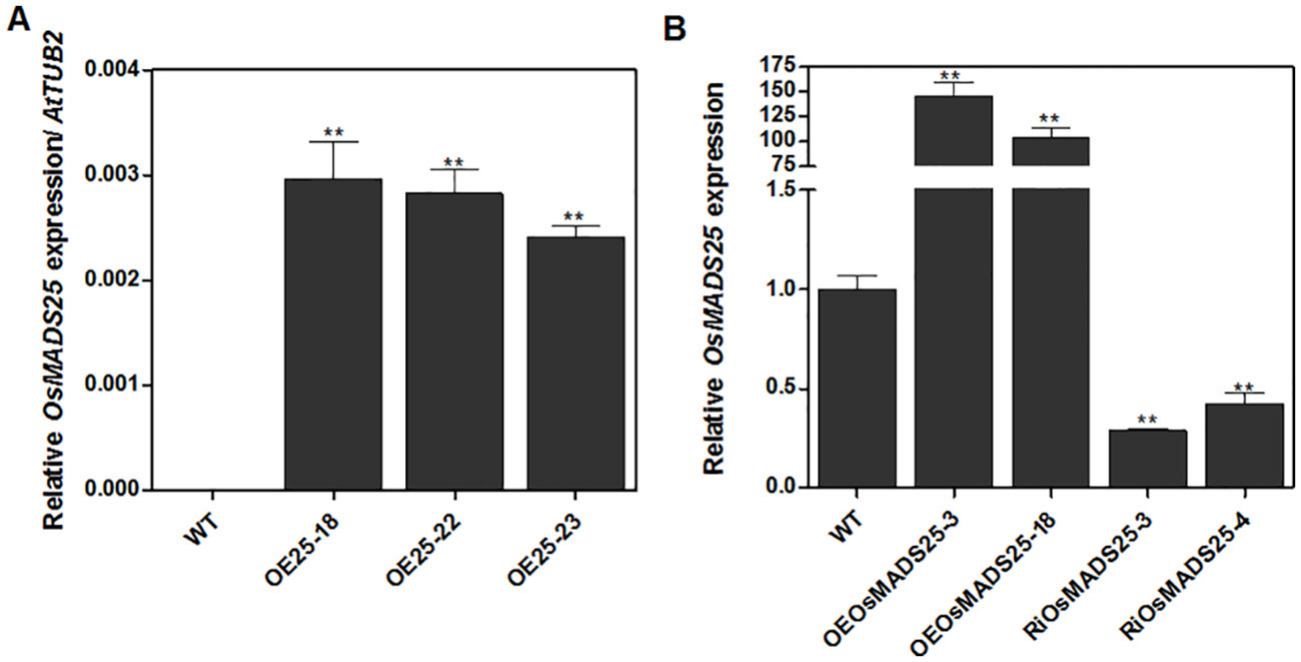
Relative expression levels of *OsMADS25* in *35S*::*OsMADS25* lines (*Arabidopsis*), *actin*::*OsMADS25* lines (rice) and *Ri*:*OsMADS25* lines (rice). (A) Seeds of *35S*::*OsMADS25* lines and the wild type were germinated and grown on MS medium for 10 d and the seedlings were transferred to the soil. After two-week growth, the rosette leaves were harvested, and the mRNA level of *OsMADS25* was performed by real-time RT-PCR. *TUB2* gene was used as a control. Error bars represent SD. (B) Two-week-old rice seedlings of both wild type and transgenic lines were grown hydroponically in complete nutrient solution were harvested. qRT-PCR was used to assay the abundance of *OsMADS25* mRNA in extracts of root RNA and *OsActin* was used as the reference. A Student’s t-test was used to analyze the significant difference between treatments at the probability of 5% (* p< 0.05) or 1% (**, P< 0.01).

**Fig 3:**
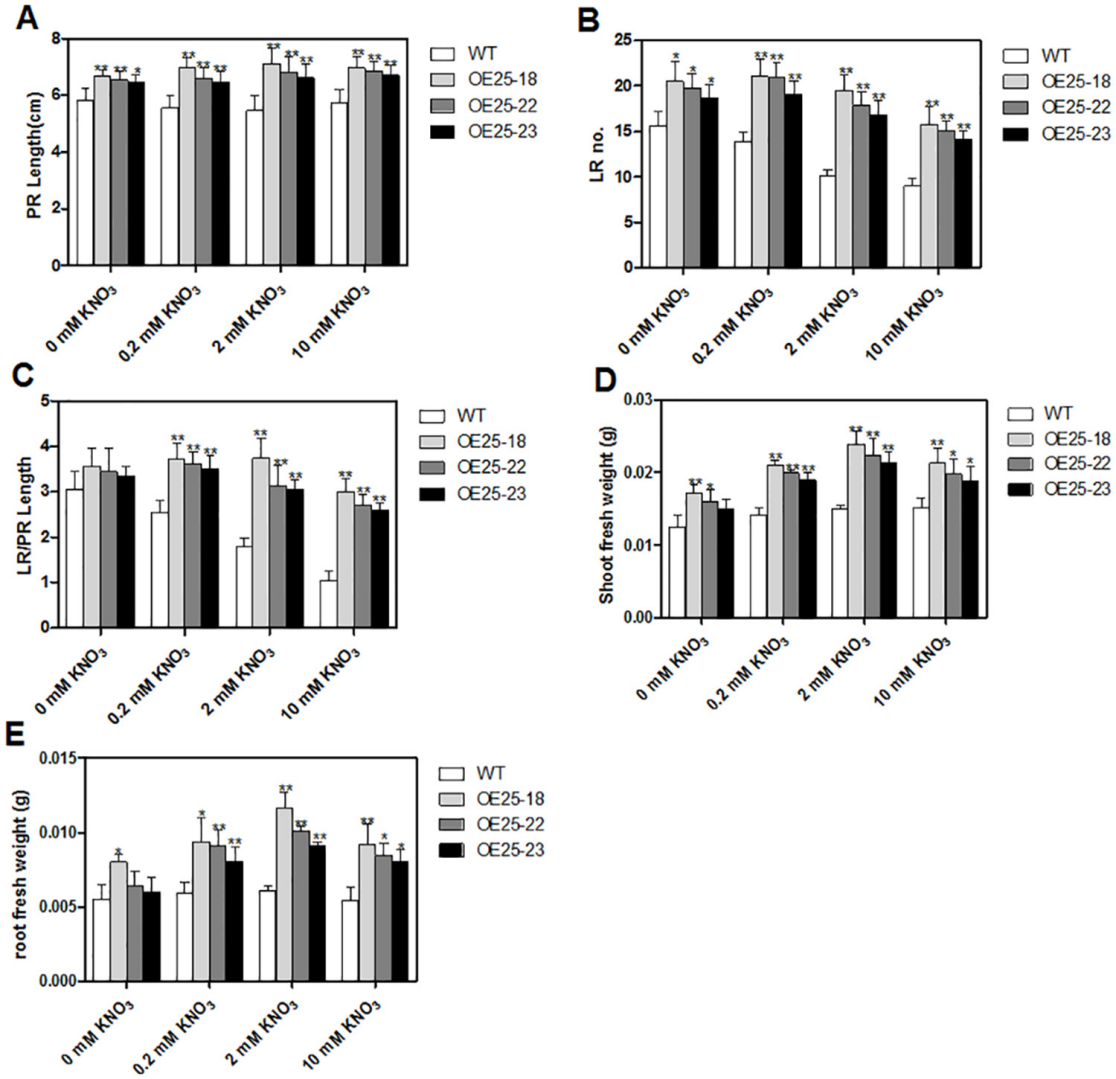
Effect of the NO_3-_ supply on root growth of three *OsMADS25*-overexpressing *Arabidopsis* lines (OE25-18, OE25-22 and OE25-23). Surface-sterilized seeds were sown in 10×10 cm rectangular Petri dishes on medium without nitrate and 7-d-old seedlings were transferred to fresh plates containing various concentrations of nitrate. Images were taken at different time intervals for measurement of root parameters. Errors indicate standard deviation (SD; n = 12). A Student’s t-test was employed to calculate the significant difference between treatments at the probability of 5% (* p< 0.05) or 1% (**, P< 0.01).

**Fig 4:**
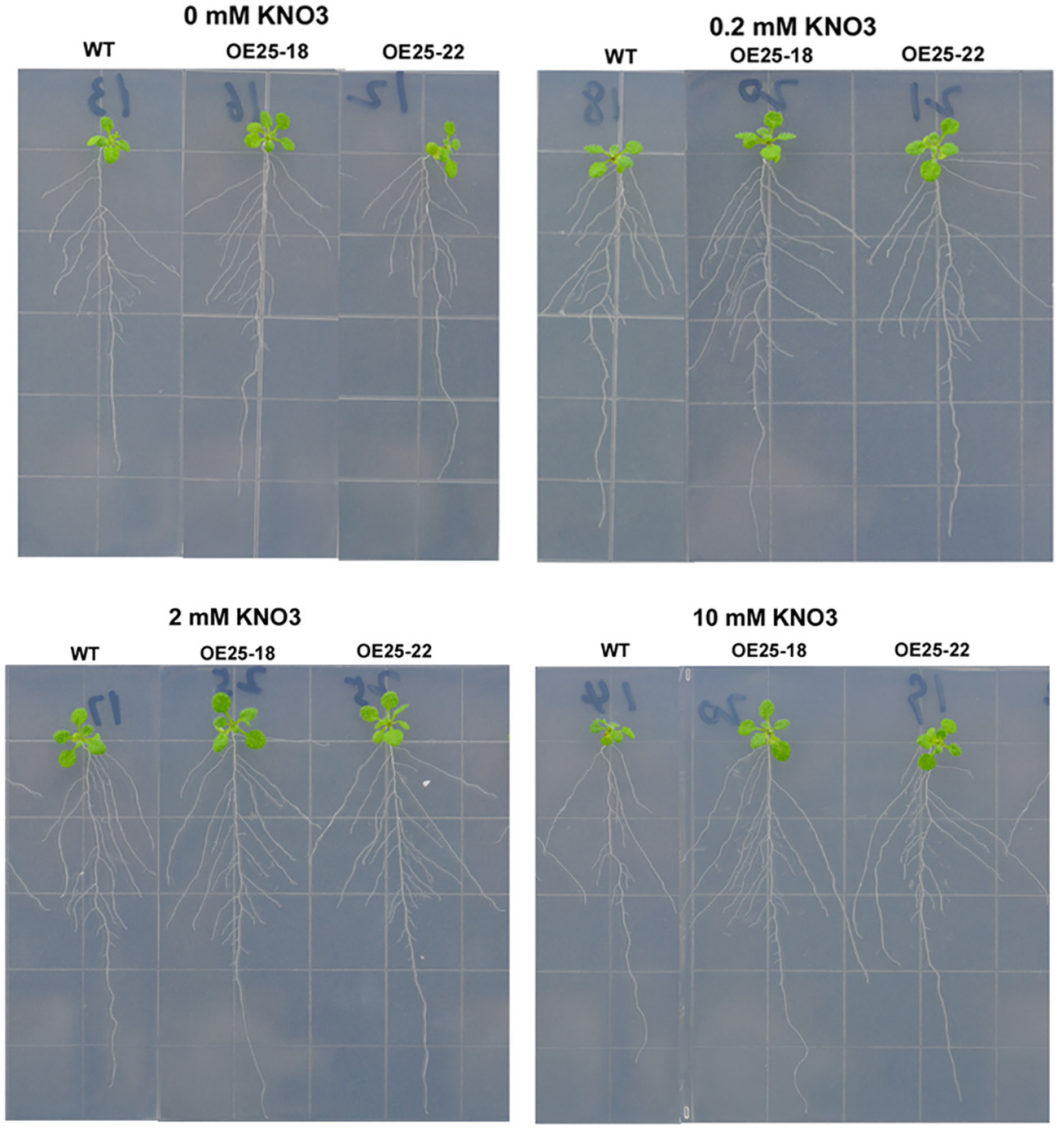
Images showing the phenotype of seedlings of Col-0 and the *OsMADS25*-overexpressing lines (OE25-18 and OE25-22) grown on vertical agar plates with different concentrations of nitrate. Seeds were germinated and grown on vertical agar plates in the absence of NO_3_^-^. 7-day-old seedlings were transferred to fresh plates consist of: 0 mM KNO_3_, 0.2 mM KNO_3_, 2 mM KNO_3_ or 10 mM KNO_3_ as N source. Images were taken at different time intervals for measurement of root parameters: 16 d (no NO_3_^-^), 14 d (0.2 and 2 mM NO_3_^-^), and 13 d (10 mM NO_3_^-^).

Since *OsMADS25* is not only closely related to *ANR1* but is also inducible by nitrate [38, 39], we investigated its possible role in NO_3_^-^ regulation of root architecture, initially using *Arabidopsis* as a model system. We created *35S*::*OsMADS25* overexpression transgenic lines and selected three representative transgenic lines that showed significantly higher *OsMADS25* expression level in comparison to wild type (Fig 2A). Seedlings of wild type Col-0 and *OsMADS25*-overexpressing lines (OE25-18, OE25-22 and OE25-23) were cultured on vertically orientated agar plates in the absence of NO_3_^-^ and when 7 d-old they were transferred to fresh plates containing a range of NO_3_^-^ concentrations. In addition to nitrate, 1 mM glutamine was also included in all treatments as a background N source so that N deficiency did not become a problem at the lower NO_3_^-^ concentrations. Because increasing NO_3_^-^ concentrations accelerate the rate of seedling development, we attempted to minimize this effect by the comparisons of root growth and branching by imaging the seedlings at 16 d after transfer (0 mM KNO_3_), 14 d (0.2 or 2 mM KNO_3_), 13 d (10 mM KNO_3_). In addition, LR length was expressed per unit primary root length to minimize effects that might arise from differences in the rate of PR growth [31]. The mean lengths of the Col-0 PRs (in cm ± SD) at the time of imaging were 5.82±0.69 (no NO_3_^-^), 5.54±0.43 (0.2 mM NO_3_^-^), 5.47±0.56 (2 mM NO_3_^-^), 5.72±0.68 (10 mM NO_3_^-^). In the absence of NO_3_^-^, *OsMADS25*-overexpressing lines (OE25-18, OE25-22 and OE25-23) showed significantly increases in PR length and LR number but no significant effect on LR length per unit PR length (Figs 3A–3C and 4). In the presence of NO_3_^-^, particularly at the higher concentrations (2 mM and 10 mM), overexpression of *OsMADS25* showed much stronger significant increase in both LR number and LR length/unit PR length (over 150% at 10 mM NO_3_^-^).

Overexpression of *OsMADS25* in *Arabidopsis* significantly increased shoot and root fresh weight in the presence and absence of NO_3_^-^, but this positive effect was much stronger in the presence of NO_3_^-^ (Fig 3D and 3E).

**Fig 5:**
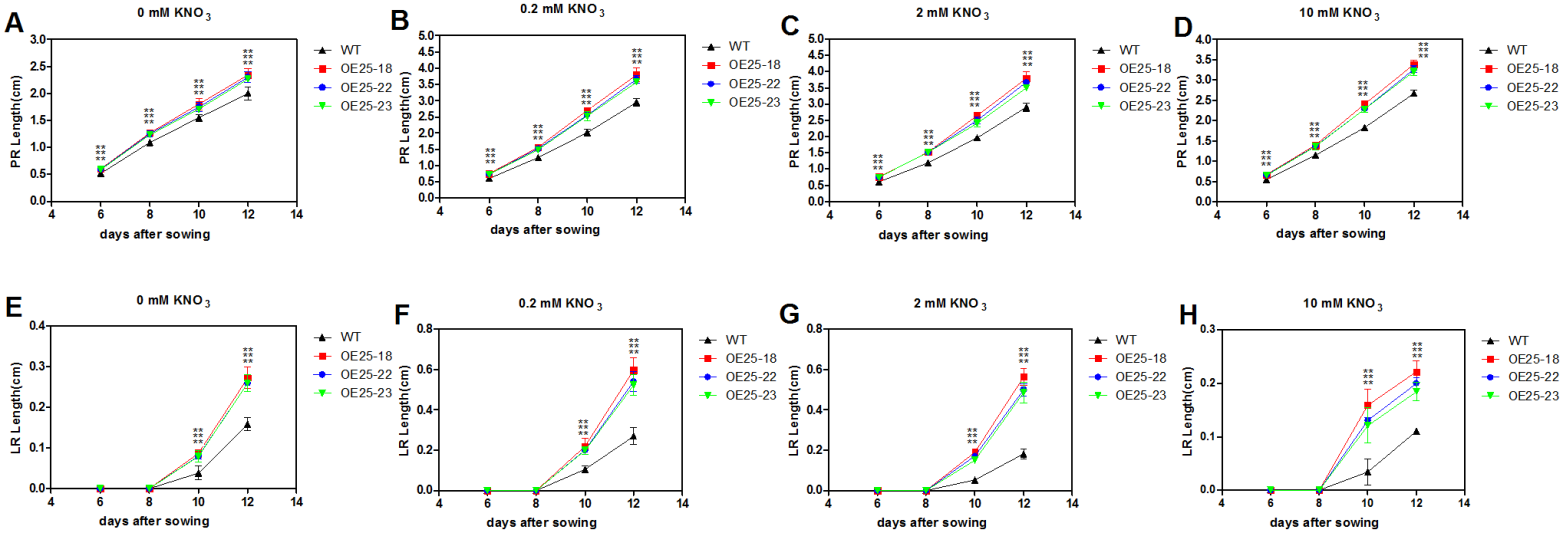
Effect of the NO_3-_ supply on early seedling development of three *OsMADS25*-overexpressing *Arabidopsis* lines (OE25-18, OE25-22 and OE25-23). (A to D) The primary root length of Col-0 and *OsMADS25*-overexpressing *Arabidopsis* lines (OE25-18, OE25-22 and OE25-23) under four different concentrations of nitrate. (E to H) First LR length of Col-0 and *OsMADS25*-overexpressing *Arabidopsis* lines (OE25-18, OE25-22 and OE25-23) under four different concentrations of nitrate. First LR was the first lateral root that emerged and extended horizontally from the primary root. Errors indicate standard deviation (SD; n = 30). A Student’s t-test was used to calculate the significant difference between treatments at the probability of 5% (* p< 0.05) or 1% (**, P< 0.01).

To investigate whether *OsMADS25* overexpression in *Arabidopsis* affected early seedling development, transgenic lines (OE25-18, OE25-22 and OE25-23) were germinated and grown on medium containing four different concentrations of nitrate. Primary root length and the first lateral root length were determined from images taken at different intervals after sowing. As shown in Fig 5, *OsMADS25*-overexpressing lines (OE25-18, OE25-22 and OE25-23) showed significantly greater PR length and the first LR length at the early seedling development stage in comparison to the wild type Col-0 control and this effect was significantly enhanced in the presence of nitrate.

### *OsMADS25* regulates root development through the NO_3_^-^ regulation in rice

**Fig 6:**
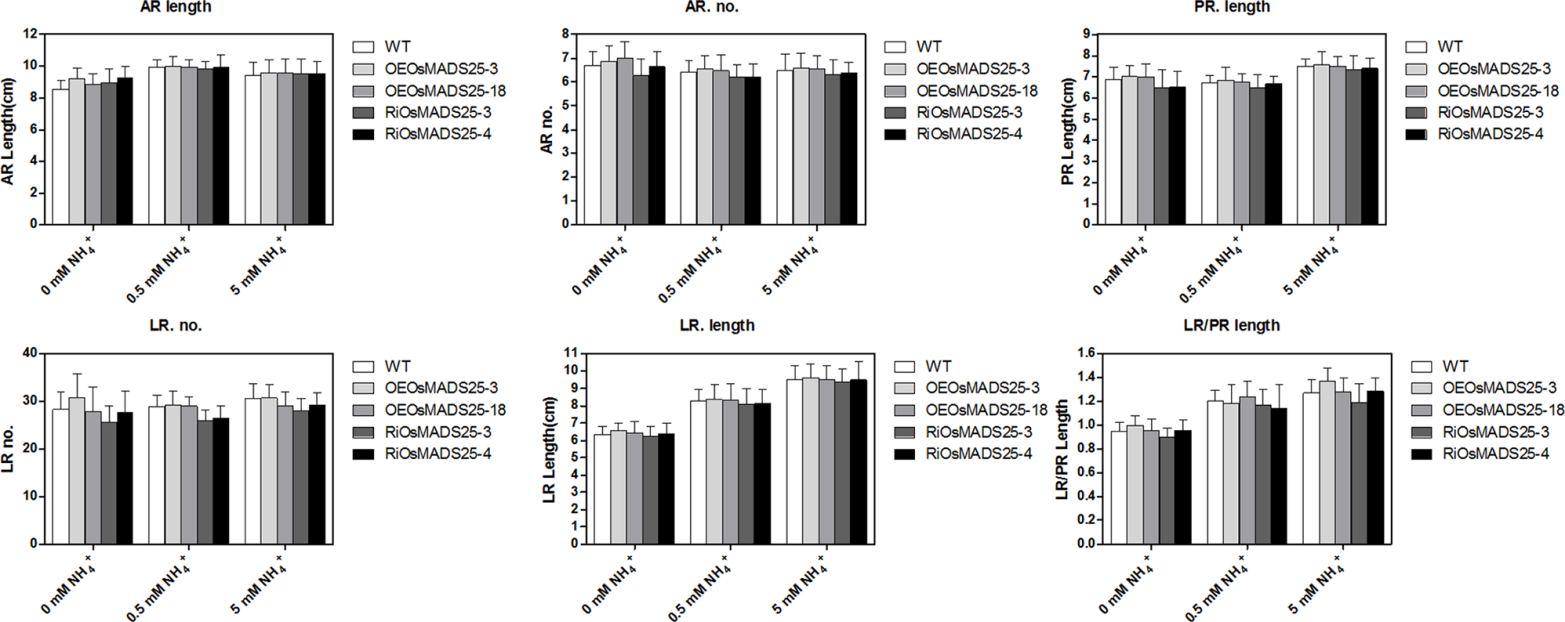
Effect of the ammonium supply on root growth of the *OsMADS25*-overexpressing lines and *OsMADS25*-interferring lines. The root phenotype of 7 d-old wild type and transgenic rice seedlings grown in 0.5 mM ammonium or 0 mM ammonium and 8 d-old seedlings grown in 5 mM ammonium was determined. OEOsMADS25-3 and OEOsMADS25-18 are two overexpressing lines and RiOsMADS25-3 and RiOsMADS25-4 are two RNAi lines. Errors indicate standard deviation (SD; n = 15–20). AR, adventitious root; LR, lateral root; PR, primary root; LR/PR length, LR length per unit PR length. A Student’s t-test was employed to calculate the significant difference between treatments at the probability of 5% (* p< 0.05) or 1% (**, P< 0.01).

Previous studies reported that the expression of *OsMADS25* was significantly regulated by NH_4_^+^ [38], we further investigated whether *OsMADS25* regulates primary and lateral root development in response to ammonium. We tested the effects of *OsMADS25* overexpression and its RNAi lines on root architecture under different concentrations of ammonium in rice. As shown in Fig 6, the numbers or length of adventitious roots, the PR length and the numbers or length of lateral roots were not significantly affected by either up- or down-regulation of *OsMADS25* in the presence or absence of ammonium in comparison to the control line.

**Fig 7:**
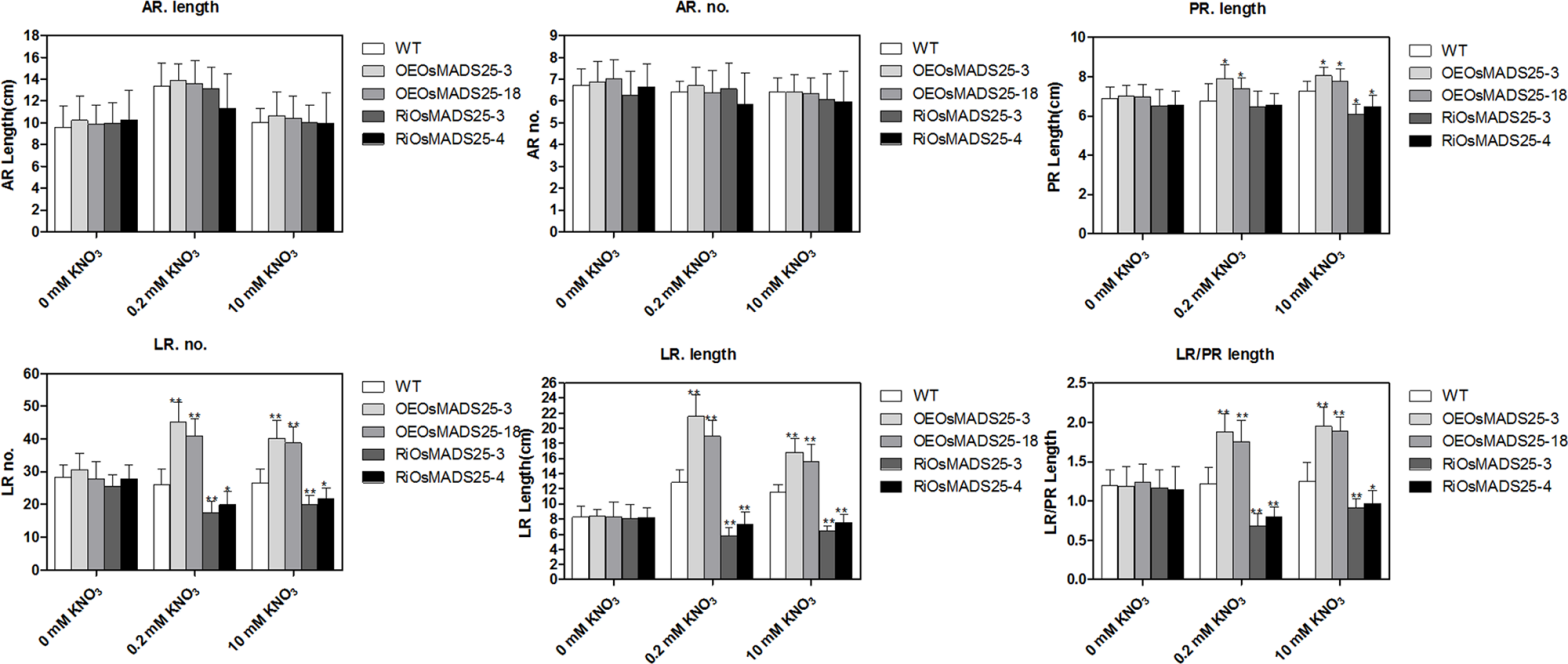
Effect of *OsMADS25* overexpression and its down-regulation by RNAi on rice root development. The root phenotype of 7 d-old wild type and transgenic rice seedlings grown in 0.2 mM nitrate or zero nitrate and 8 d-old seedlings grown in 10 mM nitrate was determined. OEOsMADS25-3 and OEOsMADS25-18 are two overexpressing lines and RiOsMADS25-3 and RiOsMADS25-4 are two RNAi lines. Errors indicate standard deviation (SD; n = 15–30). AR, adventitious root; LR, lateral root; PR, primary root; LR/PR length, LR length per unit PR length. A Student’s t-test was employed to calculate the significant difference between treatments at the probability of 5% (* p< 0.05) or 1% (**, P< 0.01).

**Fig 8:**
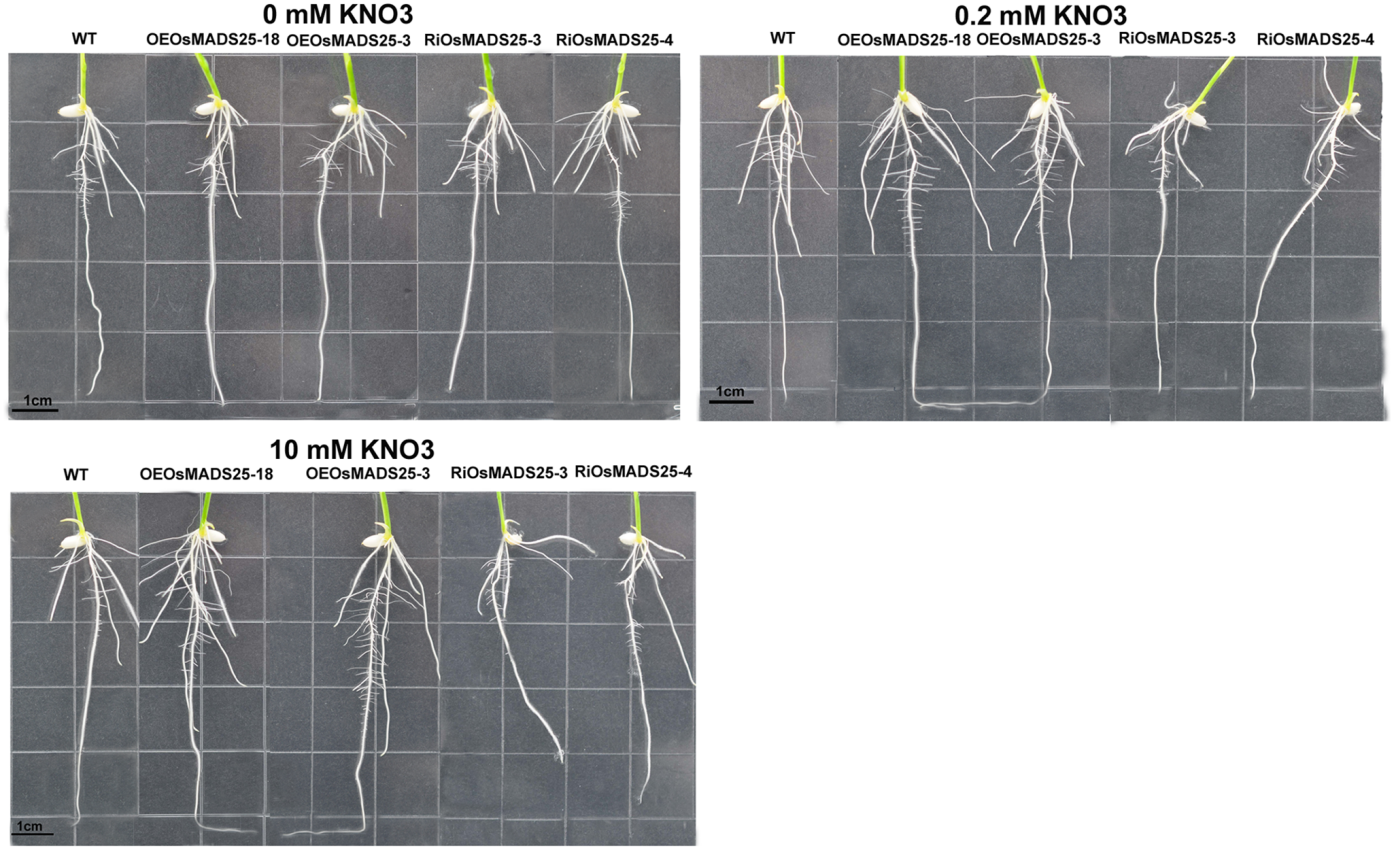
Images showing the phenotype of seedlings of wild type, the *OsMADS25*-overexpressing lines (OEOsMADS25-3 and OEOsMADS25-18) and *OsMADS25*-interferring lines (RiOsMADS25-3 and RiOsMADS25-4) grown under different concentrations of nitrate. Seeds were germinated and grown on vertical agar plates in 0.2 mM nitrate or zero nitrate and 8 d-old seedlings grown in 10 mM nitrate.

Since *OsMADS25* overexpression was able to promote LR growth and development in *Arabidopsis* (Fig 3), thus, we also want to know whether overexpression of *OsMADS25* in rice would produce the similar effect. We created *actin*::*OsMADS25* overexpression transgenic lines and obtained fifteen independent transgenic lines, from which we chose two representative transgenic lines (OEOsMADS25-3 and OEOsMADS25-18) and these two lines both showed significantly higher *OsMADS25* expression levels compared to wild type (Fig 2B). We also generated sixteen *OsMADS25*-interrupting lines and chose two typical transgenic lines (RiOsMADS25-3 and RiOsMADS25-4) which significantly suppressed the expressions of *OsMADS25* (Fig 2B). Homozygous seeds of overexpressing lines (OEOsMADS25-3 and OEOsMADS25-18) and RNAi lines (RiOsMADS25-3 and RiOsMADS25-4) and the wild type were germinated and grown on agar plates for 8 d on 10 mM NO_3_ or for 7 d on either 0.2 mM NO_3_ or no nitrate. As shown in Figs 7 and 8, the LR lengths (per unit PR length) of the OEOsMADS25-3 and OEOsMADS25-18 lines were significantly greater than those of the wild type, while those of RiOsMADS25-3 and RiOsMADS25-4 lines were significantly shorter under both high nitrate and low nitrate. The same effects, but on a smaller scale, were seen in the cases of PR length (about 10% increase and 15% decrease), LR number (about 20% increase and 20% decrease) and lateral root length (about 20% increase and 25% decrease) in the presence of nitrate, but the numbers or length of adventitious roots (AR) were not significantly affected by either up- or down-regulation of *OsMADS25*. No significant differences between wild type and transgenic lines were seen in the absence of nitrate. These results indicated that *OsMADS25* positively regulates the primary and lateral root growth in NO_3_^-^-regulation pathway in rice.

### Effect of *OsMADS25* overexpression and its down-regulation by RNAi on nitrate accumulation and shoot growth in rice

**Fig 9:**
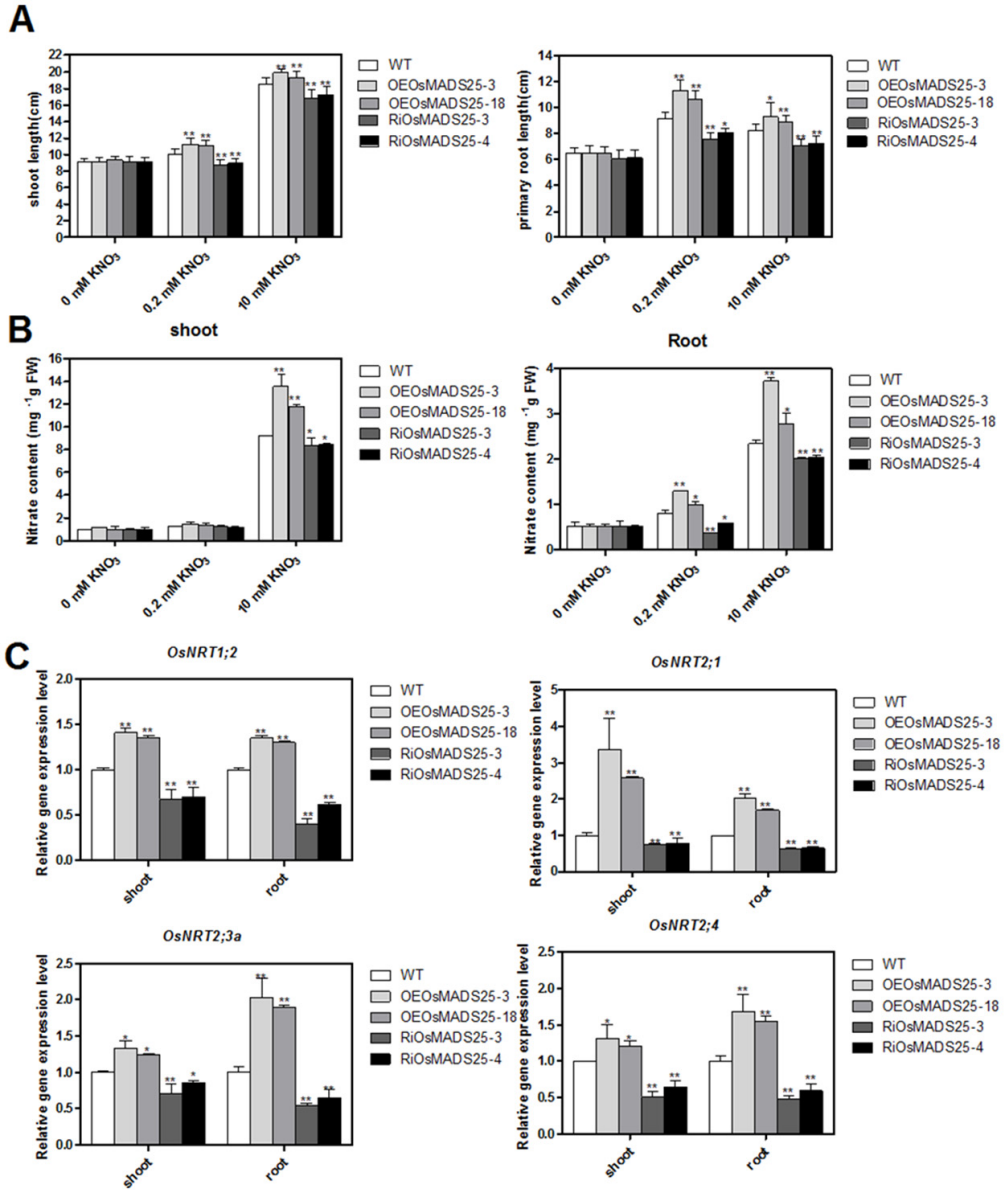
Effect of overexpression and down-regulation of *OsMADS25* on nitrate accumulation and expression of nitrate transporter genes in rice. Rice seedlings were grown in hydroponic cultures at low (0.2 mM) and high (10 mM) rates of KNO_3_ supply for 14d. (A) Lengths of shoots and primary roots. Error bars indicate standard deviation (SD; n = 20). (B) Nitrate content of in shoots and roots of wild type, *OsMADS25*-overexpressed (OEOsMADS25-3 and OEOsMADS25-18) and *OsMADS25*-down-regulated plants (RiOsMADS25-3 and RiOsMADS25-4) grown in high (10 mM KNO_3_) and low (0.2 or 0 mM KNO_3_) nitrate concentration conditions. FW, fresh weight. Error bars indicate standard error (SD; n = 12). (C) Roots and shoots of 14-d-old rice seedlings grown on 10 mM KNO_3_ were collected and qRT-PCR reactions were performed. Expression was normalized to that of *OsActin* mRNA. Significant difference was analyzed at the probability of 5% (* p< 0.05) or 1% (**, P< 0.01) by student’s t- test. Error bars indicate standard deviation (SD; n = 12).

**Fig 10:**
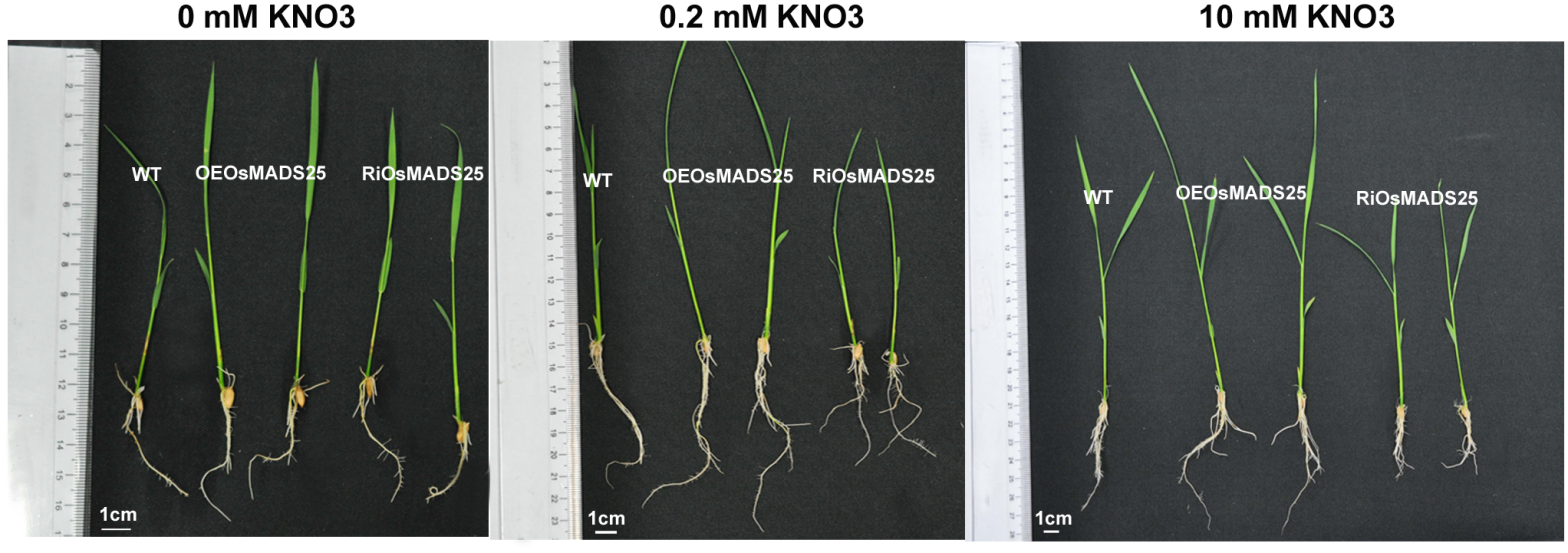
Images showing the phenotype of seedlings of wild type, the *OsMADS25*-overexpressing lines (OEOsMADS25-3 and OEOsMADS25-18) and *OsMADS25*-interferring lines (RiOsMADS25-3 and RiOsMADS25-4) grown under different concentrations of nitrate. Rice seedlings were grown in hydroponic cultures at 0 mM, 0.2 mM and 10 mM KNO_3_ supply for 14d.

To examine the effect of over-expressing and down-regulating the expression of *OsMADS25* on nitrate content and plant growth, Homozygous seeds of both wild type and transgenic plants (OEOsMADS25-3, OEOsMADS25-18, RiOsMADS25-3 and RiOsMADS25-4) were germinated and seedlings grown hydroponically for 14 d in medium containing 0, 0.2 or 10 mM KNO_3_. As shown in Figs 9A and 10, in the presence of nitrate, both shoot and primary root growth in OEOsMADS25-3 and OEOsMADS25-18 plants were significantly increased compared with the wild type, while down-regulation of *OsMADS25* produced the opposite effect. However, no significant differences were observed in these parameters in the absence of nitrate. As seen in Fig 9B, at 10 mM NO_3_^-^, *OsMADS25*-overexpressing plants showed significantly higher nitrate content in the shoot and root while *OsMADS25*-interferringing plants produced significantly lower nitrate content in shoot and root than the wild plants. However, at 0.2 mM NO_3_^-^ supply, the nitrate content in the root not in the shoot of *OsMADS25*-overexpressing lines were significantly increased and nitrate content in root of *OsMADS25* down-regulating plants was significantly decreased in comparison to the WT plants and there were no significant differences between these lines when nitrate was excluded from the medium.

As the increased nitrate content in the *OsMADS25*-overexpressing lines and decreased nitrate accumulation in the *OsMADS25*-interfering plants under 10 mM KNO_3_, we investigated the expressions of four nitrate transporter genes. As shown in Fig 9C, the mRNA abundance of four nitrate transporter genes (*OsNRT1;2*, *OsNRT2;1*, *OsNRT2;3a* and *OsNRT2;4*) were significantly decreased in the both shoots and roots of RiOsMADS25-3 and RiOsMADS25-4 lines compared with the wild type, while over-expression of *OsMADS25* significantly increased the gene expressions of these four transporters.

## Discussion

### *OsMADS25* may regulate plant growth and development and nitrate accumulation through NO_3_^-^ signaling pathway

The *ANR1* MADS box gene has previously been identified as having a key role in the regulation of LR development by external nitrate [26, 31]. Five *ANR1*-like genes have been identified in rice: *OsMADS23*, *OsMADS25*, *OsMADS27*, *OsMADS57* and *OsMADS61* [33]. Four of these (*OsMADS23*, *OsMADS27a*, *OsMADS27b*, and *OsMADS57*) are miR444a targets [32, 33, 35, 36], although only the first three of these are reported to be expressed in roots [37, 39]. It has been reported that miR444a acts as a negative regulator of the NO_3_^-^-signaling pathway to modify LR development, leading to the suggestion that it acts by controlling the expression of its *ANR1*-related targets in rice [37]. However the existence of additional targets of miR444a leaves open the possibility that other miR444a targets could be involved [35, 36]. In this study, we observed that overexpression of *OsMADS25* significantly promoted LR and PR growth in rice seedlings at early developmental stages, while interference with *OsMADS25* expression had the opposite effect and that these responses were strongest in the presence of nitrate (Fig 7). These findings are similar to what was observed in *ANR1*-overexpressing lines of *Arabidopsis*, except that *ANR1* overexpression specifically affected the LRs. In addition, we also found that there were no significant differences between wild type and transgenic seedlings under various concentrations of ammonium in rice (Fig 6). Previous studies reported that nitrate could act as a nutrient source as well as a signal to regulate gene expression, plant growth and development [5, 37, 52]. There observations indicate that the effect of altered *OsMADS25* expression on root architecture was not accounted for nutrient regulation but for nitrate signaling. These results confirmed that *OsMADS25* is a key transcriptional factor that controls root growth and development in rice and *Arabidopsis* by NO_3_^-^. Although miR444a-overexpression in rice affected adventitious root development [37], *OsMADS25*-overexpressing in rice had no significant effect on adventitious root development, which may indicate that they regulate rice root development in a different way (Fig 7).

In addition to its role in LR growth, the involvement of *ANR1* in shoot growth and nitrate accumulation has been investigated in *Arabidopsis* [31]. Previous results showed that shoot fresh weight was increased in *ANR1*-overexpressing lines, with evidence that this was likely to be a secondary effect of the larger root system [31]. Here we found that both shoot and PR growth were decreased in *OsMADS25*-downregulated rice lines compared with the wild type (Fig 9) and that the RNAi lines showed a significant reduction in nitrate accumulations and in expressions of nitrate transporters (Fig 9). Previous studies have indicated that *ANR1* acts as a positive regulator to promote the expression of the *NRT2*.*1* - a high-affinity nitrate transporter in *Arabidopsis* [30]. Therefore it seems likely that *OsMADS25* is also a positive regulator of nitrate accumulation in rice and that the nitrate content increases in shoot and root in the overexpressing line confirmed this role. In conclusion, this study characterized a novel MADS-box transcription factor *OsMADS25*, which plays a key role in regulation primary and lateral root development in rice. Overexpression of *OsMADS25* significantly increased lateral and primary root growth in the presence of high (10 mM) or low concentration of nitrate (0.2 mM). These results suggested that *OsMADS25* might positively regulate root and shoot development through NO_3_^-^-regulation pathway in rice. Furthermore, overexpression of *OsMADS25* altered the expressions of nitrate transporter genes, thus leading to increase nitrate accumulation under 10 mM NO_3_^-^. Further study will be needed to explore the molecular mechanism in details to explain the role of *OsMADS25* in regulating shoot and root development through NO_3_^-^-signaling pathway.
